# Paroxysmal Atrial Fibrillation in the Course of Acute Pulmonary Embolism: Clinical Significance and Impact on Prognosis

**DOI:** 10.1155/2017/5049802

**Published:** 2017-02-09

**Authors:** Agnieszka Krajewska, Katarzyna Ptaszynska-Kopczynska, Izabela Kiluk, Urszula Kosacka, Robert Milewski, Jacek Krajewski, Wlodzimierz Jerzy Musial, Bozena Sobkowicz

**Affiliations:** ^1^Department of Cardiology, Medical University of Białystok, Białystok, Poland; ^2^Department of Cardiology, Procardia, Augustów, Poland; ^3^Department of Statistics and Medical Informatics, Medical University of Białystok, Białystok, Poland; ^4^Department of Anaesthesiology and Intensive Care, Medical University of Białystok, Białystok, Poland

## Abstract

The relationship and clinical implications of atrial fibrillation (AF) in acute pulmonary embolism (PE) are poorly investigated. We aimed to analyze clinical characteristics and prognosis in PE patients with paroxysmal AF episode.* Methods*. From the 391 patients with PE 31 subjects with paroxysmal AF were selected. This group was compared with patients with PE and sinus rhythm (SR) and 32 patients with PE and permanent AF.* Results*. Paroxysmal AF patients were the oldest. Concomitant DVT varies between groups: paroxysmal AF 32.3%, SR 49.5%, and permanent AF 28.1% (*p* = 0.02). The stroke history frequency was 4.6% SR, 12.9% paroxysmal AF, and 21.9% permanent AF (*p* < 0.001). Paroxysmal AF comparing to permanent AF and SR individuals had higher estimated SPAP (56 versus 48 versus 47 mmHg, *p* = 0.01) and shorter ACT (58 versus 65 versus 70 ms, *p* = 0.04). Patients with AF were more often classified into high-risk group according to revised Geneva score and sPESI than SR patients. In-hospital mortality was lower in SR (5%) and paroxysmal AF (6.5%) compared to permanent AF group (25%) (*p* < 0.001).* Conclusions*. Patients with PE-associated paroxysmal AF constitute a separate population. More severe impairment of the parameters reflecting RV afterload may indicate relation between PE severity and paroxysmal AF episode. Paroxysmal AF has no impact on short-term mortality.

## 1. Introduction

Atrial fibrillation (AF) is the most common age-related, sustained cardiac arrhythmia. It accounts for 4% of cases of arrhythmia in the population older than 60 years and for 8% of cases in patients older than 80 years [1]. AF adversely affects the prognosis mainly because of thromboembolic complications such as stroke, development of heart failure, or progression of preexisting heart failure. The association between AF and acute PE is complex and not fully elucidated. The two conditions have some common risk factors, such as obesity, heart failure, myocardial infarction, and hypertension [2]. The risk of AF as well as pulmonary embolism (PE) increases with age. It has not been unequivocally explained whether the presence of AF in patients with PE affects their prognosis. So far, it has not been well established whether AF can lead to episodes of PE due to right-side intracardiac thrombi formation. Moreover, data concerning the prognostic significance of paroxysmal AF in patients with PE are sparse [3]. Paroxysmal AF may occur as a consequence of PE due to acute right ventricular (RV) systolic overload and subsequent right atrial dilation. Therefore, we decided to explore the hypothesis of whether paroxysmal AF could be a sign of PE severity and thus a marker of worse prognosis. We aimed to analyze the significance of paroxysmal AF that develops in the course of acute PE and to evaluate clinical characteristics of these patients, performance of the two prognostic scores for PE, and an impact of paroxysmal AF on short- and long-term all-cause mortality compared to patients in sinus rhythm (SR) and permanent AF.

## 2. Materials and Methods

**Figure 1:**
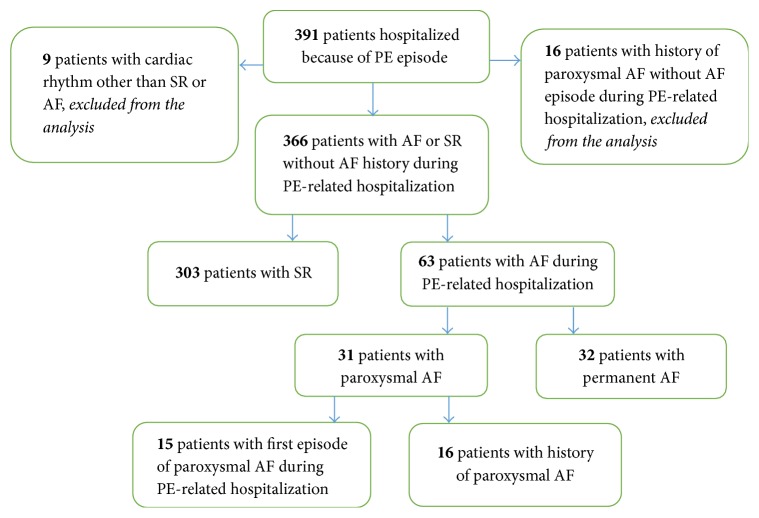
Study flow chart. The characteristics of the study population according to the heart rhythm. AF: atrial fibrillation; PE: pulmonary embolism; SR: sinus rhythm.

The study cohort consisted of 391 consecutive patients with primary diagnosis of acute PE. Patients were aged 18 years or older and were hospitalized in the Department of Cardiology at University Hospital in Białystok, Poland, from January 1, 2004, to December 31, 2013. Their medical records were retrospectively analyzed. In 344 patients (88%), the diagnosis of PE was established by thoracic computed tomography angiography. In the remaining 47 patients (12%), it was confirmed on the basis of pulmonary ventilation-perfusion scintigraphy or echocardiography. As a standard procedure during index hospitalization, color duplex ultrasound of the lower extremity was performed to assess the presence of deep vein thrombosis (DVT). The study population was then divided into three groups (Figure 1):

- the sinus rhythm (SR) group included patients in SR throughout the hospital stay;
- the paroxysmal AF group included patients with one or more episodes of paroxysmal AF documented by electrocardiogram (ECG) at any time during index hospitalization. This group included patients in SR on admission who developed paroxysmal AF during hospital stay and patients with AF on admission with sustained SR during subsequent hospital stay;
- the permanent AF group included patients with the diagnosis of permanent AF confirmed by ECG.

There were no patients with valvular AF in the study cohort. In addition, patients with AF in history who did not develop AF during hospital stay were excluded.

On admission, demographic and clinical characteristics, including symptoms, hemodynamic profile, oxygen saturation, length of hospital stay, risk factors for PE, and comorbidities, were evaluated and compared between the groups. In addition, the probability of PE was evaluated retrospectively using the revised version of the Geneva score rule [4, 5], and the Simplified Pulmonary Embolism Severity Index (sPESI) was calculated retrospectively. The following laboratory parameters were also measured on admission: complete blood count, levels of troponin I, D-dimer, and estimated glomerular filtration rate (eGFR, using the Modification of Diet in Renal Disease formula).

Transthoracic echocardiography was performed within 24 hours of admission, and the following parameters were analyzed: left ventricular ejection fraction (LVEF) estimated by visual assessment, left atrial dimensions, presence of RV contractility disturbances, systolic pulmonary artery pressure determined using the simplified Bernoulli equation (SPAP = 4*V*maxTR^2^ + RAP, where *V*maxTR is the maximal velocity of tricuspid regurgitant jet and RAP is the estimated right atrial pressure), pulmonary artery acceleration time, and presence of thrombi in the right heart cavities or in the pulmonary artery.

Finally, a standard 12-lead surface electrocardiogram was recorded to analyze the leading rhythm and the presence of tachycardia (>100 beats/min).

### 2.1. Study Outcomes

The primary outcome of the study was all-cause mortality. Data on all-cause in-hospital mortality were retrieved from medical records. The long-term outcome of the study cohort was retrieved from a national death registry database provided by the Polish Ministry of Home Affairs. A censored date of October 13, 2015, was determined to allow a minimum follow-up of 20 months for living patients (range, 20–178 months). In addition, we decided to analyze one-year survival.

The study protocol was approved by local ethics committee.

### 2.2. Statistical Analysis

In statistical analysis, categorical variables were compared using the chi-square test of independence. Normality of distribution was evaluated by the Kolmogorov-Smirnov test with the Lilliefors correction and the Shapiro-Wilk test. There was nonnormal distribution of continuous variables. The quantitative parameters were characterized by a median and quartiles and the nominal parameters using percentages. The nonparametric Kruskal-Wallis test with post hoc test was used to compare quantitative variables without normal distribution between the three groups.

Survival was estimated using the Kaplan-Meier method for each rhythm group. Differences between survival curves in the three groups were evaluated using the chi-square test.

For all tests, a *p* value of less than 0.05 was considered statistically significant. Statistical analysis was performed using the Statistica 12.0 software (StatSoft, Inc., Tulsa, USA).

## 3. Results

The clinical characteristics, outcome, laboratory parameters, and risk score profiles of the study population stratified according to SR, paroxysmal AF, and permanent AF are shown in Tables 1–3. Of 391 patients with confirmed diagnosis of PE, we identified 63 individuals (16%) with AF. Thirty-one patients (7.9%) were classified as having paroxysmal AF, and 15 of them (48%) reported a history of AF. In 32 patients (8.2%), permanent AF was confirmed. Nine patients were excluded from the study as having cardiac rhythm other than sinus or AF (Figure 1).

Patients with paroxysmal AF were a median of 13 years older than patients in SR and a median of 4 years older than those with permanent AF (Table 1). There were no differences in sex distribution or in clinical symptoms on admission between the three groups (Tables 1 and 2).

The frequency of stroke in history significantly increased from 4.6% in patients with SR to 12.9% in those with paroxysmal AF and to 21.9% in those with permanent AF (Table 1).

Concomitant DVT during index hospitalization occurred significantly less often in patients with paroxysmal AF compared with the SR group. However, the proportion of patients with DVT was the lowest in patients with permanent AF (Table 2). The hemodynamic profile on admission revealed a higher prevalence of tachycardia (>100 beats/min) in both AF groups than in the SR group, with the highest proportion of patients with tachycardia in the group with paroxysmal AF (Table 2).

In the analysis of laboratory parameters, the only difference between the three groups was shown for eGFR: it was significantly lower in patients with paroxysmal as well as permanent AF as compared with those with SR (Table 3). Among echocardiographic parameters, patients with paroxysmal AF had the highest median value of estimated systolic pulmonary artery pressure and the shortest median pulmonary artery acceleration time (Table 3). On the other hand, patients with paroxysmal AF had significantly higher median values of left ventricular ejection fraction and smaller left atrial diameter compared with the permanent AF group (Table 3). Patients with AF also showed a tendency to have a higher rate of right heart thrombosis. Furthermore, this rate tended to be higher in patients with permanent than in those with paroxysmal AF, although the difference was not significant.

The retrospective analysis of the probability of PE, using the revised version of the Geneva score rule, showed significant differences between the three groups. Patients with both types of AF were more likely to be classified into the high-probability group than patients with SR. The high-probability group included more patients with paroxysmal than with permanent AF (Table 2). The same trend was shown for the sPESI. Eighty-six percent (86.2%) of patients with paroxysmal AF group had a sPESI of 1 or higher, compared with 82.6% of patients with permanent AF and 56.2% of those with SR (Table 2).

**Figure 2:**
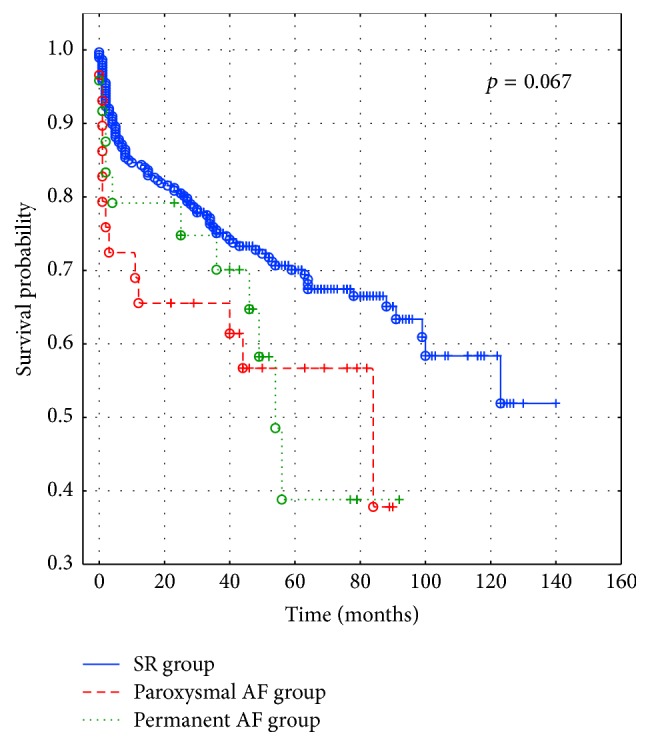
The Kaplan-Meyer curves in PE patients comparing survival between three groups: the sinus rhythm (SR) group, paroxysmal atrial fibrillation (paroxysmal AF) group, and permanent atrial fibrillation (permanent AF) group.

Regarding in-hospital mortality, it was significantly lower in patients with SR and paroxysmal AF (5% and 6.5%, resp.) compared to patients with permanent AF (25%) (Table 1). The results of the Kaplan-Meier survival analysis are presented in Figure 2. There was a trend towards worse survival in patients with paroxysmal AF in comparison with those with permanent AF and particularly those with SR, although the differences were not significant (*p* = 0.067).

## 4. Discussion

In this study, we found that, among patients with an acute episode of PE, those who develop paroxysmal AF on admission or during hospital stay have a different clinical presentation than those in SR or with permanent AF. We performed a detailed analysis of our baseline data, including the results of an echocardiographic examination of patients with paroxysmal AF and those with permanent AF. The data suggest that paroxysmal AF may be a sign of PE severity and may affect long-term prognosis. To date, no studies have been published that would focus specifically on paroxysmal AF in patients with PE. Regarding the analyzed variables, patients with paroxysmal AF are situated distinctly in between patients with SR and permanent AF. Interestingly, there were no significant differences between the three groups in terms of most comorbidities, risk factors, and symptoms of PE on admission, as well as the length of hospital stay.

The hypothesis that PE may provoke AF is grounded on a pathophysiological basis. Sudden RV systolic overload results in an increase of right atrial pressure, which in turn leads to atrial arrhythmias. In the present study, in an echocardiographic examination, patients with paroxysmal AF demonstrated the indirect signs of RV overload such as the shortest artery acceleration time and the highest estimated systolic pulmonary artery pressure compared with patients in SR or in those with permanent AF. Patients with paroxysmal AF also showed a trend towards the highest troponin I concentrations, although the differences with the other groups were not significant. Such results have never been demonstrated before.

In our study population the prevalence of AF was higher than in general population [1] and paroxysmal AF comprised nearly half of cases of AF. Depending on the inclusion criteria [3, 6, 7], the prevalence of AF in patients with PE was reported between 9% and 44%. Some investigators included both patients with AF on admission, as shown on an electrocardiogram, as well as those with a history of AF without AF at index hospitalization in a single AF group [6, 8]. Most authors did not differentiate AF into paroxysmal, persistent, and permanent [3, 6, 8–10]. There have been only a few studies investigating the relationship between PE and paroxysmal AF [6, 9–11]. In one study [5] the proportion of patients with paroxysmal AF was comparable. In another study paroxysmal AF was identified in 13% of participants [10]. The difference in the prevalence of paroxysmal AF was probably due to the design of the study, which investigated the quality of oral anticoagulation in a cohort of patients with a history of venous thromboembolism (VTE) at any time of their life. The problem of inefficient anticoagulation has been raised previously [12]. There were no data concerning the acuteness of a VTE episode and a temporal sequence of VTE and AF events.

AF may be not only a consequence of PE but also a risk factor for PE. It induces the prothrombotic state due to activation of the coagulation cascade and platelets [13]. Lack of atrial contraction results in blood stasis and possibility of thrombus formation in both atria, particularly in their appendages [14, 15]. Data concerning the association between PE, AF, right heart thrombus formation, and prognosis have been recently reported [5, 16]. Surprisingly, the authors did not find an association between AF and RHT. We observed a tendency to the higher prevalence of RHT both in patients with paroxysmal AF and in those with permanent AF, but the difference did not reach significance.

Another indirect argument supporting the hypothesis about a causal relationship between AF and subsequent PE is the observation concerning lower frequency of concomitant DVT in patients with unprovoked PE [9–11]. In one study both paroxysmal AF and nonparoxysmal AF were associated with the increased risk of VTE (particularly PE), with the same statistical significance. In another study permanent AF was more common than paroxysmal AF in patients with isolated PE [10]. In our study in patients with SR, concomitant DVT was detected significantly more often than in patients with paroxysmal AF but the lowest frequency of concomitant DVT was discovered in patients with permanent AF.

Data on the effect of AF on outcome in patients with acute PE are rare and unequivocal. In some studies, negative impact on mortality was demonstrated [3, 6]. Some investigators did not find any association between AF in patients with PE and prognosis of these patients [10, 17]. Also, it is not known whether AF is an independent risk factor for mortality or whether it occurs as a consequence of PE severity or the presence of comorbidities. Furthermore, in the majority of studies concerning patients with PE, the effect of AF on survival was analyzed without differentiation of AF patterns.

There are several possible explanations why patients with paroxysmal AF in our study had worse echocardiographic parameters reflecting RV afterload and the highest proportion of an sPESI score of 1 or higher both indicating worse prognosis, but without impact on in-hospital mortality. One possibility is that both the signs of RV dysfunction on echocardiogram and sPESI help identify low-risk patients more accurately than high-risk patients [18–20]. Another possibility is that, in patients with SR and paroxysmal AF, there were two important prognostic makers whose median values were within the normal range: LVEF and eGFR [21].

Another important issue that emerged in our study relates to the usefulness of risk scores in PE patients with paroxysmal AF. We decided to verify the Geneva score rule, calculated retrospectively on admission, as well as the sPESI score. Their prognostic value in long-term follow-up was demonstrated among patients with confirmed PE [22, 23]. In our study the high-probability group included the highest percentage of patients with paroxysmal AF compared with the other groups. This probably resulted from older age and the prevalence of tachycardia (>100 beats/min) on admission in patients with paroxysmal AF. Despite the results of both scales, the in-hospital mortality rate of patients with paroxysmal AF was rather low, in contrast to patients with permanent AF in whom the rate was high.

The sPESI score was initially developed for 30-day risk assessment in patients with PE [24]. In our PE patients with paroxysmal AF, sPESI showed better prognostic value for long-term survival than for in-hospital outcome. Again it is possible that the presence of tachycardia typical for a paroxysmal episode of AF may constitute a confounding variable. This may mean that, in patients with PE complicated by a paroxysmal episode of AF, the current scales may overestimate short-term risk.

Our study has several strengths and limitations. The main limitation is the retrospective analysis of the patient's data. As a result, we have incomplete information concerning prior anticoagulation in patients with AF. On the other hand, the strengths of our study include a large number of patients from the same center, well-validated in-hospital data, and long-term follow-up.

## 5. Conclusions

Our study showed that individuals with paroxysmal AF constitute a separate population of patients than patients with PE and SR or those with permanent AF. Worse echocardiographic parameters reflecting RV afterload may indicate a causal association between the severity of PE and an episode of paroxysmal AF. This factor has significance but only for long-term prognosis. Further studies on a larger population of patients with PE are needed to determine the prognostic significance of AF types in patients with PE as well as the accuracy of PE risk scales in patients with different AF patterns.

## Figures and Tables

**Table 1:** Characteristics and outcome of the patients with pulmonary embolism stratified for the presence of the sinus rhythm (SR), paroxysmal atrial fibrillation (paroxysmal AF), and permanent atrial fibrillation (permanent AF).

	SR (*n* = 303), Me (Q1–Q3) or %	Paroxysmal AF (*n* = 31), Me (Q1–Q3) or %	Permanent AF (*n* = 32), Me (Q1–Q3) or %	*p* value
Age, years	64 (49–76)^a,b^	78 (69–82)^b^	74 (67–79)^a^	<0.001
Males	44.2%	32.3%	43.8%	0.44
Obesity (BMI ≥ 30)	37.9%	41.7%	21.7%	0.27
Overweight (BMI 25–30)	25.0%	16.7%	26.1%	0.65
Current smoker	13,3%	7.7%	16.7%	0.62
Ex-smoker	23.8%	7.7%	25%	0.17
Length of hospital stay, days	9.0 (7–12)	10.0 (8–13)	9.0 (4–12)	0.13
*Comorbidities*				
Cardiovascular disease	12.9%	13.4%	15.6%	0.57
Arterial hypertension	54.8%	58.1%	56.3%	0.9
Diabetes	14.5%	16.1%	12.5%	0.92
Chronic obstructive pulmonary disease	5.3%	9.7%	3.1%	0.49
History of stroke	4.6%	12.9%	21.9%	<0.001
*Mortality*				
In-hospital mortality	5%	6.5%	25%	<0.001

Me (Q1–Q3) or %: data presented as a median and interquartile range or a percent of the group.

^a^ *p* value < 0.01; ^b^*p* value < 0.001.

BMI: body mass index (kg/m^2^).

**Table 2:** Comparison of the admission clinical parameters, Geneva risk score results, and sPESI score values in patients with sinus rhythm (SR), paroxysmal atrial fibrillation (paroxysmal AF), and permanent atrial fibrillation (permanent AF).

	SR (*n* = 303), Me (Q1–Q3) or %	Paroxysmal AF (*n* = 31), Me (Q1–Q3) or %	Permanent AF (*n* = 32), Me (Q1–Q3) or %	*p* value
*PE symptoms*				
Syncope	19.0%	26.7%	10%	0.25
Chest pain	30.0%	36.7%	30%	0.75
Dyspnea	86.6%	96.7%	79.3%	0.14
Hemoptysis	2.0%	0	6.3%	0.2
Cough	8.3%	6.7%	16.7%	0.28
*PE* associated with DVT	49.5%	32.3%	28.1%	0.02

*Risk factors*				
Immobilization	19.1%	19.4%	34.4%	0.13
Malignancy	17.8%	9.7%	18.8%	0.5
Pregnancy/delivery	3.3%	0	0	0.34
Recurrent PE	6.6%	0	3.1%	0.26

The revised Geneva risk score: clinical probability				0.04
Low	17.3%	16.1%	12.5%	
Intermediate	76.1%	64.5%	68.8%	
High	6.6%	19.4%	18.8%	
sPESI score ≥ 1	56.2%	86.2%	82.6%	<0.001

*Hemodynamic profile on admission*				
Heart rate, beats per minute	89.5 (78–103)^a^	99 (78–124)	101 (81–122)^a^	0.01
Tachycardia (>100 beats/minute)	32.0%	54.8%	50%	0.08
Systolic blood pressure, mmHg	130 (115–145)	125 (106–145)	126 (109–142)	0.6
Oxygen saturation, %	95 (92–97)	95 (90–97)	95 (90–96)	0.17

Me (Q1–Q3) or %: data presented as a median and interquartile range or a percent of the group.

^a^ *p* value *p* = 0.05.

DVT: deep vein thrombosis; PE: pulmonary embolism.

**Table 3:** Comparison of the baseline laboratory and echocardiographic parameters in patients with sinus rhythm (SR), paroxysmal atrial fibrillation (paroxysmal AF), and permanent atrial fibrillation (permanent AF).

	SR (*n* = 303), Me (Q1–Q3) or %	Paroxysmal AF (*n* = 31), Me (Q1–Q3) or %	Permanent AF (*n* = 32), Me (Q1–Q3) or %	*p* value
*Biochemical parameters*				
eGFR, ml/min/1.73 m^2^	76 (59–93)^c,d^	63 (51–83)^d^	53 (33–79)^c^	<0.001
Troponin I, ng/ml	0.066 (0.01–0.4)	0.11 (0.046–0.42)	0.036 (0.008–0.34)	0.3
D-dimer, ng/ml	5.6 (3–12.0)	10.6 (3.5–19.9)	11 (4.2–15.1)	0.3
Hemoglobin, g/dl	12.7 (11.4–14)	13.2 (11.4–14.7)	12.6 (10.7–15)	0.58

*Echocardiography*				
LVEF, %	60 (50–60)^c^	55 (50–60)^b^	48 (30–55)^b,c^	<0.001
LA, cm	3.7 (3.3–4.0)^a,c^	3.9 (3.6–4.4)^a,b^	4.4 (4.2–5.1)^b,c^	<0.001
SPAP, mmHg	47 (37–59)^b^	56 (47–70)^b^	48 (45–59)	0.01
ACT, ms	70 (54–95)^a^	58 (51–65)^a^	65 (55–80)	0.04
RV wall contractility disturbances	58.4%	77.4%	67.9%	0.09
Thrombus in RA/RV	5.2%	10%	14.3%	0.11

Me (Q1–Q3) or %: data presented as a median and interquartile range or a percent of the group

^a^ *p* value < 0.05; ^b^*p* value < 0.01; ^c^*p* value < 0.001; ^d^*p* value *p* = 0.05.

ACT: acceleration time; GFR: glomerular filtration rate; LA: left atrium; LVEF: left ventricular ejection fraction; SPAP: systolic pulmonary artery pressure; RV: right ventricle.
